# Genetic and clinical phenotypic analysis of carney complex with external auditory canal myxoma

**DOI:** 10.3389/fgene.2022.947305

**Published:** 2022-08-23

**Authors:** Wei Wan, Liang Zeng, Hongqun Jiang, Yunyan Xia, Yuanping Xiong

**Affiliations:** ^1^ Department of Otolaryngology-Head and Neck Surgery, First Affiliated Hospital of Nangchang University, Jiangxi, China; ^2^ Jiangxi Institute of Otorhinolaryngology-Head and Neck Surgery, Jiangxi, China

**Keywords:** carney complex, PRKAR1A gene, mutation-genetics, external auditory canal myxoma, pediatrics

## Abstract

**Background:** Mutations in PRKAR1A gene can lead to Carney complex (CNC), and most CNC patients develop cardiac and cutaneous myxomas. In particular, cardiac myxomas are a common cause of mortality in CNC patients. Cutaneous myxomas of the external ear are extremely rare, and do not have any specific clinical features

**Methods:** In this retrospective study, we analyzed the clinical and genetic data of the proband and his family and fifty whole blood control samples selected from the molecular genetic database of our hospital. Whole exome DNA sequencing analysis was used to detect the mutation in the peripheral blood samples.

**Results:** The results of the clinical analysis showed the presence of spotty skin pigmentation and external auditory canal myxoma in the proband as well as in his sister and mother. Whole-exome DNA sequencing showed a novel heterozygous mutation in the PRKAR1A gene i.e., c.824_825delAG (p.Gln275Leufs*2), in the proband and his sister and mother.

**Conclusion:** In conclusion, the family members had the same autosomal dominant PRKAR1A mutation. DNA sequencing revealed a novel c.824_825delAG in exon 9 of PRKAR1A. This pathogenic mutation has not been reported previously, and may be related to the occurrence of external auditory canal myxomas and spotty pigmentation. This study broadens the genotypic spectrum of PRKAR1A mutations in CNC.

## Introduction

Carney complex (CNC) is a rare, autosomal dominant, multiple neoplasia syndrome with various clinical manifestations, including spotty skin pigmentation and myxomas (Carney et al., 1985; Carney, 1995; Briassoulis et al., 2012a). Spotty pigmentation may affect any part of the body and develops on the face, lips, and genitalia in approximately 80% of CNC children (Sandrini and Stratakis, 2003). In addition, most CNC patients develop cardiac and cutaneous myxomas, which are the most common tumors seen in infancy. In particular, cardiac myxomas are a common cause of mortality in CNC patients (Boikos and Stratakis, 2007; Espiard and Bertherat, 2013). Cutaneous myxomas of the external ear are extremely rare, and do not have any specific clinical features, except skin spots before the onset of hearing loss, deafness, and pain due to occlusion of the caused by external auditory canal occlusion. External ear myxomas in CNC, patients can be easily confused with other external auditory canal diseases, and particularly with other tumors (Ferreiro and Carney, 1994).

CNC is a common neoplasia syndrome, and associated with endocrine overactivity and multiple tumors (Stratakis et al., 1998; Stratakis et al., 2000), including pituitary adenomas, primary pigmented nodular adrenocortical disease, thyroid tumors, testicular tumors, and ovarian lesions (Stratakis et al., 1997; Stratakis et al., 1999; Pack et al., 2000; Stratakis et al., 2000; Stratakis et al., 2001). A molecular genetic study demonstrated that more than 70% of CNC patients have mutations in the *PRKAR1A* gene, which is a classic tumor suppressor gene that encodes for the 1-α regulatory subunit (R1α) of the cyclic adenosine monophosphate (cAMP)-dependent protein kinase A (PKA) (Kirschner et al., 2000; Bertherat et al., 2009). *PRKAR1A* gene mutations cause R1α loss, augment PKA activity, and enhance downstream signaling, which lead to uncontrolled tumor suppression (Kirschner et al., 2000). Moreover, myxomas, acromegaly, and other symptoms are more common in patients with exon mutations than in those with PRKAR1A defects (Bertherat et al., 2009). Horvath (Horvath et al., 2010) reported the functional effects of molecular *PRKAR1A* mutations, and described the clinical manifestations of specific mutations. Few studies have demonstrated a correlation between specific mutations and CNC manifestations. In this study, we performed genetic and clinical analyses of a Han Chinese family with suspected CNC. The study analyzed the genotype- phenotype correlation in patients CNC patients using clinical phenotyping to laid early CNC diagnosis.

## Materials and methods

The family analyzed in this study were from Jiangxi Province, China. In January 2020, two siblings presented for surgical treatment of unilateral external auditory canal lesions. A detailed medical history was obtained from the parents, the family underwent detailed physical examinations, including ear examinations by a specialist, hearing tests, and adrenal and thyroid ultrasonograms. Tissue samples were obtained from the external auditory canals of the proband and his sister for pathological analysis. Their mother also experienced symptoms of the disease. Whole bloods amples were obtained from the proband and other family members for DNA analysis. Fifty whole blood control samples were randomly selected from the molecular genetic database of our hospital. Informed consent forms was obtained from the participants. Other pathogenic variants of stapes ankylosis were ruled out.

Whole blood samples were collected using 3–5 ml of ethylenediaminetetraacetic acid as an anticoagulant. Genomic DNA from blood samples was used to construct libraries. The target genes and DNA near the shear region were enriched using a Human All Exon V4 kit (BGI Group, Shenzhen, China), and the mutations were detected using the MGISEQ-2000 sequencing platform (BGI Group). The average effective sequencing depth in the target area was ≥ 100×. The sequences were compared using the Burrows-Wheeler Aligner and UCSC Human Gene Mutation Database (HG19) reference genomes to remove duplicates. GATK was used for genotyping, adjustment of base mass values, and detection of single-nucleotide variants, insertions, deletions. The criteria for pathogenicity were based on the American College of Medical Genetics guidelines. The sequencing results were compared with the HG19, 1,000 Genome, ExAC, ESP6500, GnomAD, dbNSFP, and other databases.

## Results

**FIGURE 1:**
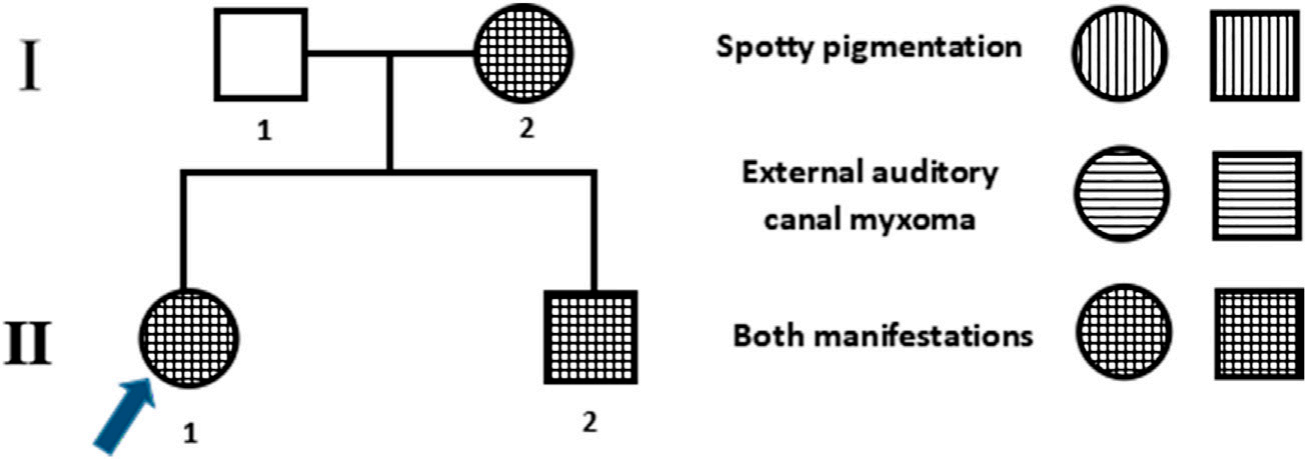
Family pedigree. Males and females are denoted by squares and circles, respectively. The arrow denotes the proband, filled symbols denote Carney complex (CNC) patients, and unfilled symbols denote unaffected individuals. Symbols with horizontal lines denote individuals with spotty pigmentation, symbols with vertical lines denote those with external auditory canal myxomas, and symbols filled with horizontal and vertical lines denote those with both manifestations.

**FIGURE 2:**
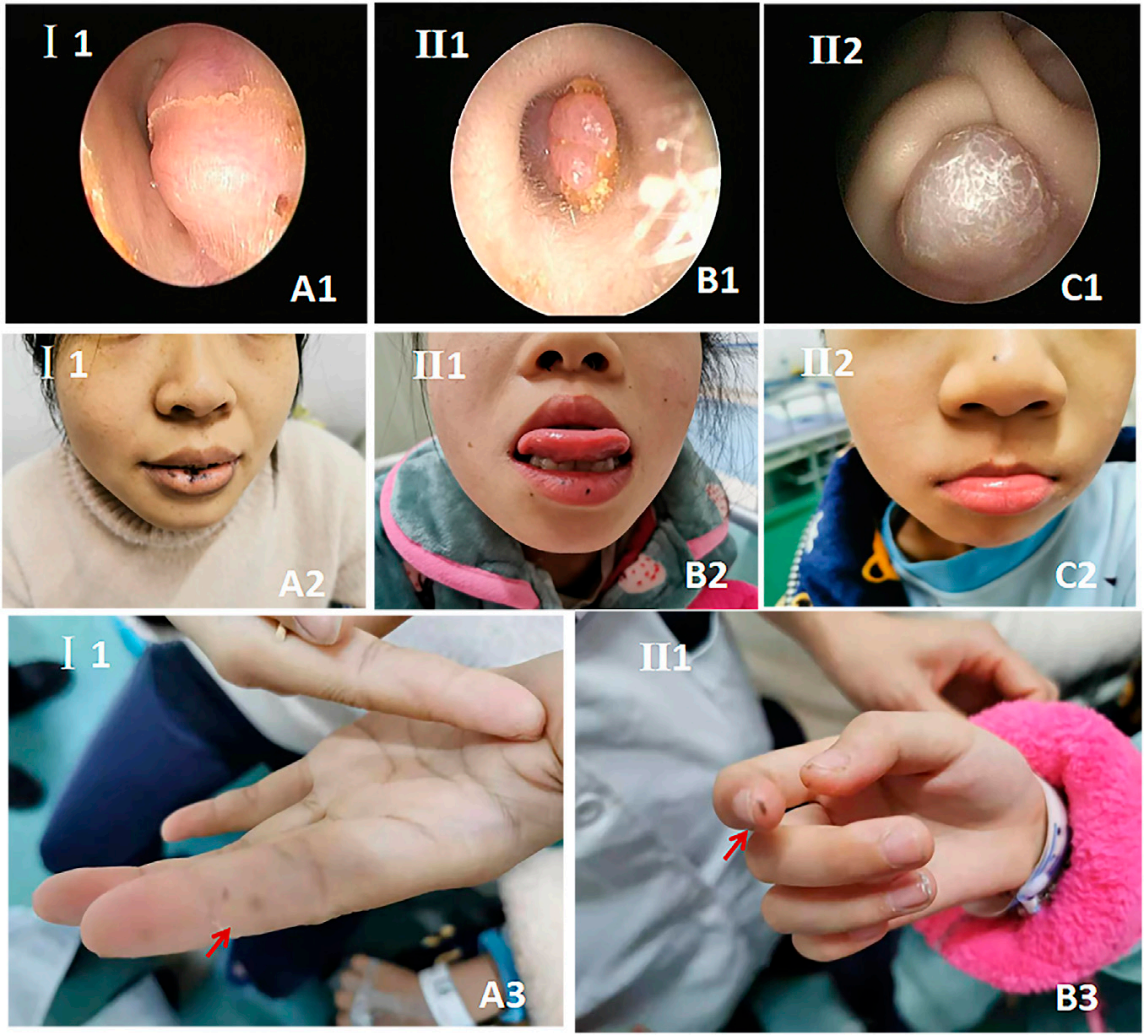
Patient characteristics and clinical manifestations. **(A1–A3)** Proband’s mother; **(B1–B3)** proband’s sister; **(C1–C2)** the proband. **(A1–C1)**. External auditory canal otoendoscopy. **(A2–C2)**. Lip and facial spotty pigmentation. **(A3,B3)**. Spotty skin pigmentation on the finger.

**FIGURE 3:**
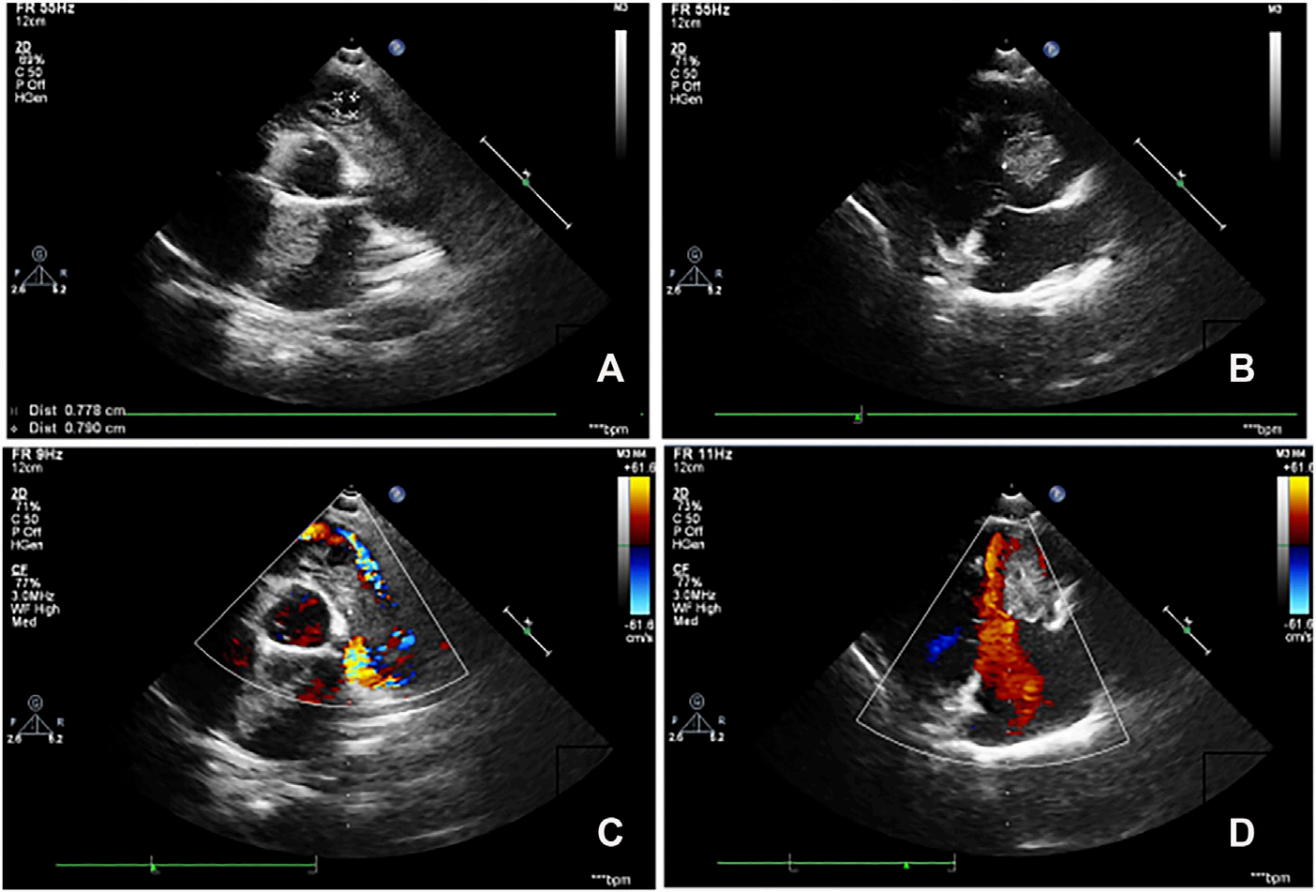
Echocardiography images of the proband. **(A,B)**. Clumpy echogenic mass in the left atrium and right ventricle. **(C,D)**. No obvious enhancement was observed on the enhanced scan.

**FIGURE 4:**
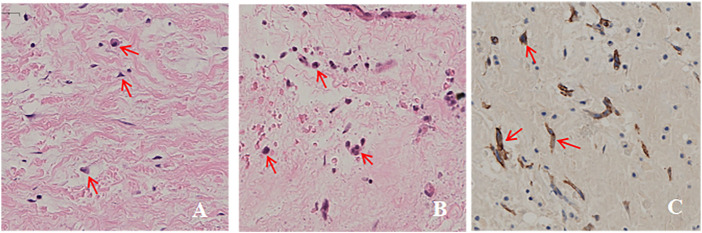
Histopathological images. **(A,B)**. Ear tumor of the proband and his sister was composed of tumor cells in a mucous matrix (hematoxylin-eosin, original magnification ×200). **(C)**. Immunohistochemical analysis of paraffin-embedded cardiac myxoma tissue from the proband showing tumor cells (brown) (200×).

Three members of the family were diagnosed with CNC, consistent with its autosomal dominant inheritance (Figure 1). The proband’s parents denied inbreeding. The proband and his sister reported progressive hearing loss over the past year, with no history of otitis media, ototoxic drug use, or noise exposure. There were no significant differences in growth and development compared to other patients and peers. Spotty pigmentation was found in the fingers, face, and lingual dorsal mucosa of the proband and his sister and mother (Figure 2). The proband and his sister had well-demarcated unilateral external auditory canal lesions. Ear computed tomography revealed slight low-density filling of the affected external auditory canal and its bony walls. Pure-tone audiometry showed ipsilateral conductive deafness. Histopathology suggested a diagnosis of external auditory canal myxoma (Figure 4). PRKAR1A DNA sequencing indicated a novel site mutation, c.824_825delAG (p.Gln275Leufs*2), in exon 9. Ultrasonography of the proband revealed a low-density msss in the left atrium and right ventricle, with no significant enhancement (Figure 3), No cardiac myxomas were found in the remaining family members on echocardiography. Adrenal and thyroid gland ultrasonography showed no obvious abnormalities. Histopathologically, the tumor samples of the proband and his sister were composed of spindle or stellate cells associated with mucous stroma and small blood vessels. Immunohistochemistry showed tumor cells, consistent with the myxoma (Figure 4).

**FIGURE 5:**
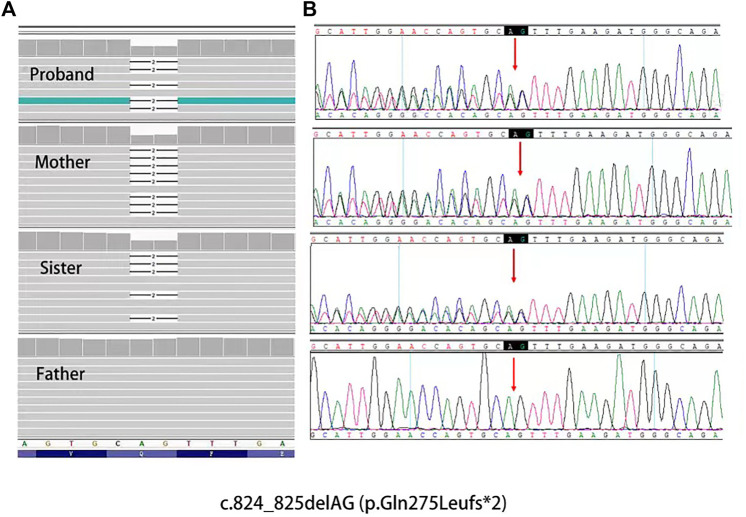
**(A)**, Mutation data of this family in Integrtive Genomics Viewer (IGV) software. **(B)**, Sanger sequencing results of PRKAR1A gene showed heterozygosity for the **(C)**824_825delAG mutation (arrow) of the family.

Whole-exome DNA sequencing showed a novel heterozygous mutation in the *PRKAR1A* gene i.e., c.824_825delAG (p.Gln275Leufs*2), in the proband and his sister and mother (Figure 5). To confirm that this was a pathogenic mutation, we screened 50 healthy controls for the *PRKAR1A* gene. None of the controls had the mutation; therefore, suggesting that it was pathogenic rather than polymorphic.

**FIGURE 6:**
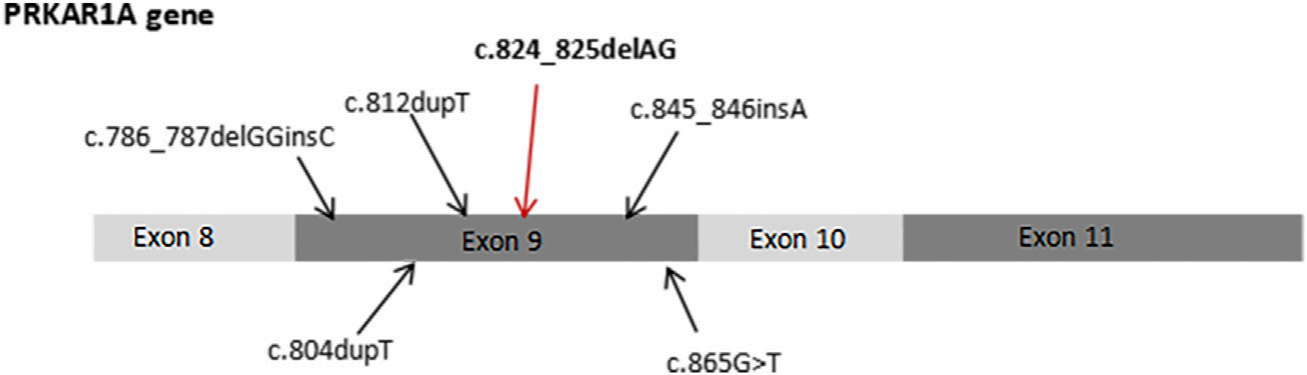
Previously reported PRKAR1A mutations in exon 9, including c.824_825delAG.

We searched the PubMed database to identify literature on the *PRKAR1A* gene. More than 140 *PRKAR1A* pathogenic mutations were identified in the *PRKAR1A* Mutation (http://PRKAR1A.nichd.nih.gov) and PRKAR1A databases, most of which were located in exons. We identified a heterozygous mutation (c.824_825delAG) not found in any large next-generation sequencing (ExAC and GnomAD) or public single nucleotide polymorphism databases (Figure 6). PROVEN was used to predict the profiles of proteins potentially generated by this mutation. We found that the mutation introduced a premature stop codon, which led to truncated translation of 277 amino acids and a truncated protein. Moreover, this mutation significantly affected secondary structure predictions of the RIα domain.

## Discussion

**FIGURE 7:**
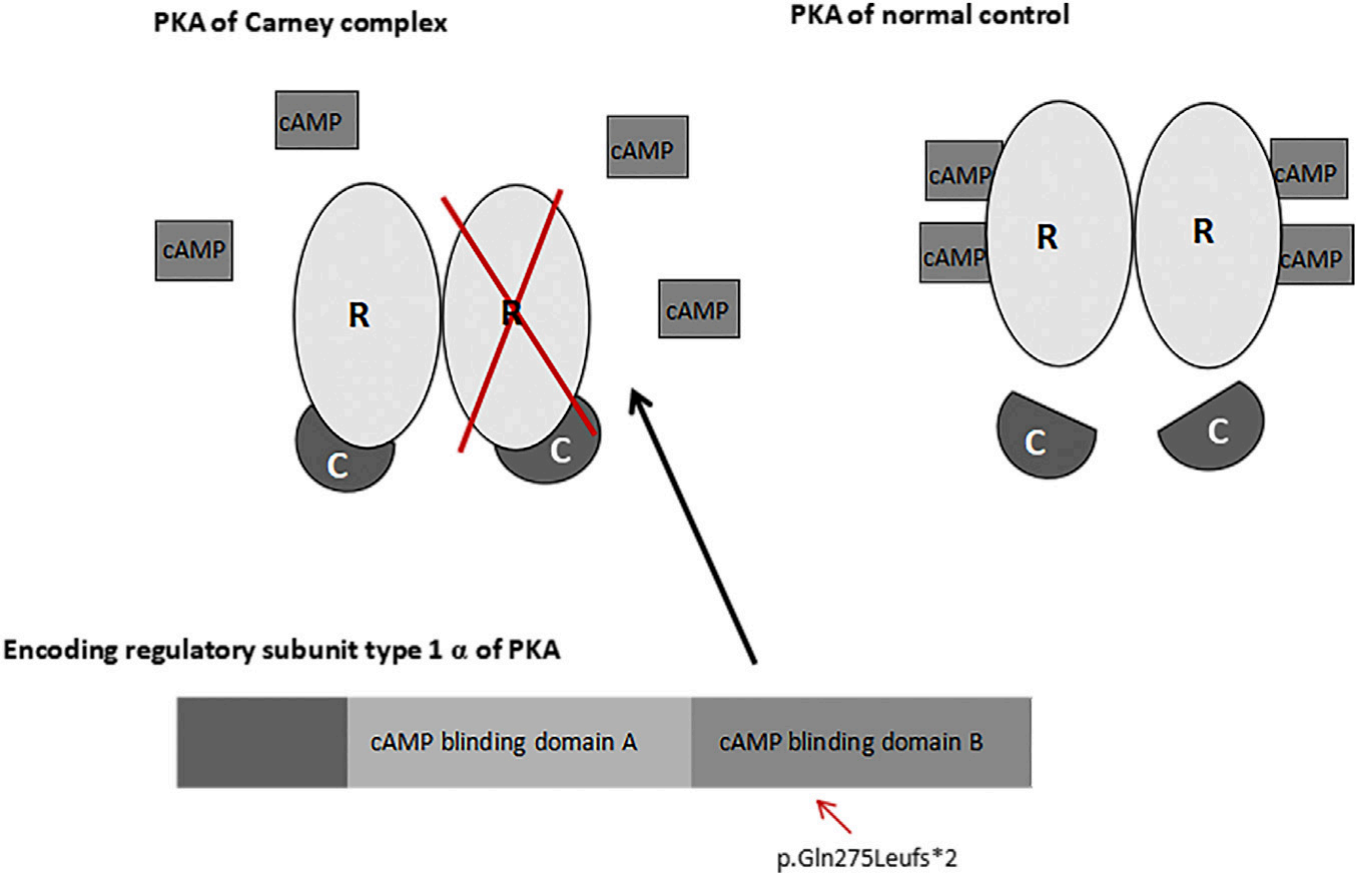
*PRKAR1A* and PKA pathways. A change in the coding protein inactivated the cAMP nucleotide binding domain B in PKA. As a result, Camp failed to bind with the regulatory subunit (R) or release the catalytic subunit (C) of PKA.

CNC is an autosomal dominant syndrome; a positive family history is present in approximately 70% of patients (Bertherat et al., 2009). The wild-type *PRKAR1A*-encoding DNA sequence contains 1,146 bases, and produces a protein containing 381 amino acids (Fu et al., 2018). Approximately 62% of CNC patients have inactivated mutations which are encoded by the PRKAR1A gene in the R1α domain of cAMP-dependent PKA (Kirschner et al., 2000; Robinson-White et al., 2003; Tsilou et al., 2004; Bertherat et al., 2009). Biochemically, inactivation of the R1α egulatory subunit enhances PKA activity and downstream signaling (Bertherat et al., 2009). CNC is heterogeneous condition involving 2p16 (CNC2) and 17q22_24 (CNC1) (Matyakhina et al., 2003). Approximately 80% of the pathogenic *PRKAR1A* variants are affected by mRNA nonsense-mediated decay of the mutant sequence, which affects *PRKAR1A* haploinsufficiency by causing deletions in the mutant protein (Bertherat et al., 2009; Bonnet-Serrano and Bertherat, 2018; Kamilaris et al., 2019). In the present study, we report a novel mutation in the mRNA coding region within exon 9 of the *PRKAR1A* gene. Based on the DNA sequencing results, we hypothesized that the deletion altered the coding protein at 275aa (Gln to Leu), which changed the amino acid at 277 to a terminating amino acid, and resulted in a truncated protein. The cAMP nucleotide binding domain B in PKA was inactivated; therefore, cAMP failed to bind with the PKA regulatory subunit and release the catalytic subunit. Consequently, regulation of the PKA catalytic subunit was lost, leading to activation of the cAMP-PKA pathway, increased mitosis and cell proliferation (Amieux and McKnight, 2002; Kamilaris et al., 2019) (Figure 7). Previous studies have demonstrated that *PRKAR1A* haploinsufficiency and overactivity may lead to eyelid myxomas (Robinson-White et al., 2003; Tsilou et al., 2004; Rothenbuhler and Stratakis, 2010; Kamilaris et al., 2019), consistent with our findings of external auditory canal myxomas.

CNC is a rare syndrome with two main manifestations verified histologically, biochemically, or by imaging. Patients with a single manifestation have an inactivating *PRKAR1A* mutation or first-degree relative with CNC (Carney et al., 1985; Kamilaris et al., 2019). Although skin pigmentation is useful for early diagnosis of CNC, it is not always observed in the early stages, and other signs are required for an accurate diagnosis (Carney et al., 1986; Ferreiro and Carney, 1994).

The most common tumors of infancy in CNC patients are cardiac and cutaneous myxomas of the eyelid, breast, ear, and external genitalia (Ferreiro and Carney, 1994). Isolated ear myxomas unrelated to CNC are rare (Palva et al., 1991), and thus diagnostically important. Most external auditory myxomas are misdiagnosed as polyps or tumors, which are amenable to simple excision. External ear myxomas have no specific histopathological features and should be differentiated from fibroepithelial polyps and papillomas, squamous papillomas, ear polyps, and hamartomas. (Ferreiro and Carney, 1994). External auditory canal myxomas are pathologically similar to cutaneous myxomas. These mucoid lesions are covered with keratinized squamous epithelial and exhibit obvious basaloid bud proliferation. The lesion consists of scattered stellate and spindle cells, with lymphocyte infiltration (Ferreiro and Carney, 1994; Rothenbuhler and Stratakis, 2010). Previous descriptions are consistent with our pathological finding.

CNC is a rare autosomal dominant disease, but the relationships among its clinical manifestations, phenotypes, and genetic profile are not clear. Most previous reports external auditory canal myxomas in CNC patients were isolated cases. Briassoulis (Briassoulis et al., 2012b) reported a myxoma behind the right ear in the 23-month-old daughter of a CNC patient. A *PRKAR1A* was detected in this patient, her sister, and mother, but only the patient had an external auditory myxomas. In the present study, external auditory myxomas were detected in early childhood in all CNC patients in this family. Therefore, we speculated that the c.824_825delAG mutation might be related to the clinical manifestations observed in this family, but this requires further validation.

In conclusion, the proband and his sister and mother had spotty pigmentation and external ear myxomas. The family members had the same autosomal dominant *PRKAR1A* mutation. DNA sequencing revealed a novel c.824_825delAG mutation in exon 9 of *PRKAR1A*. This pathogenic mutation (PVS1+PM2) has not been reported previously, and may be related to the occurrence of external auditory canal myxomas and spotty pigmentation. This study broadens the genotypic spectrum of *PRKAR1A* mutations in CNC, and may lead to a better understanding of CNC pathogenesis.

## Data Availability

The datasets presented in this study can be found in online repositories. The names of the repository/repositories and accession number(s) can be found in the article/Supplementary Material.
