# Supplementary figures and images for: Genetic and clinical phenotypic analysis of carney complex with external auditory canal myxoma
Source: Front Genet. 2022 Aug 23;13:947305. doi: 10.3389/fgene.2022.947305 (PMC9450949; doi:10.3389/fgene.2022.947305)

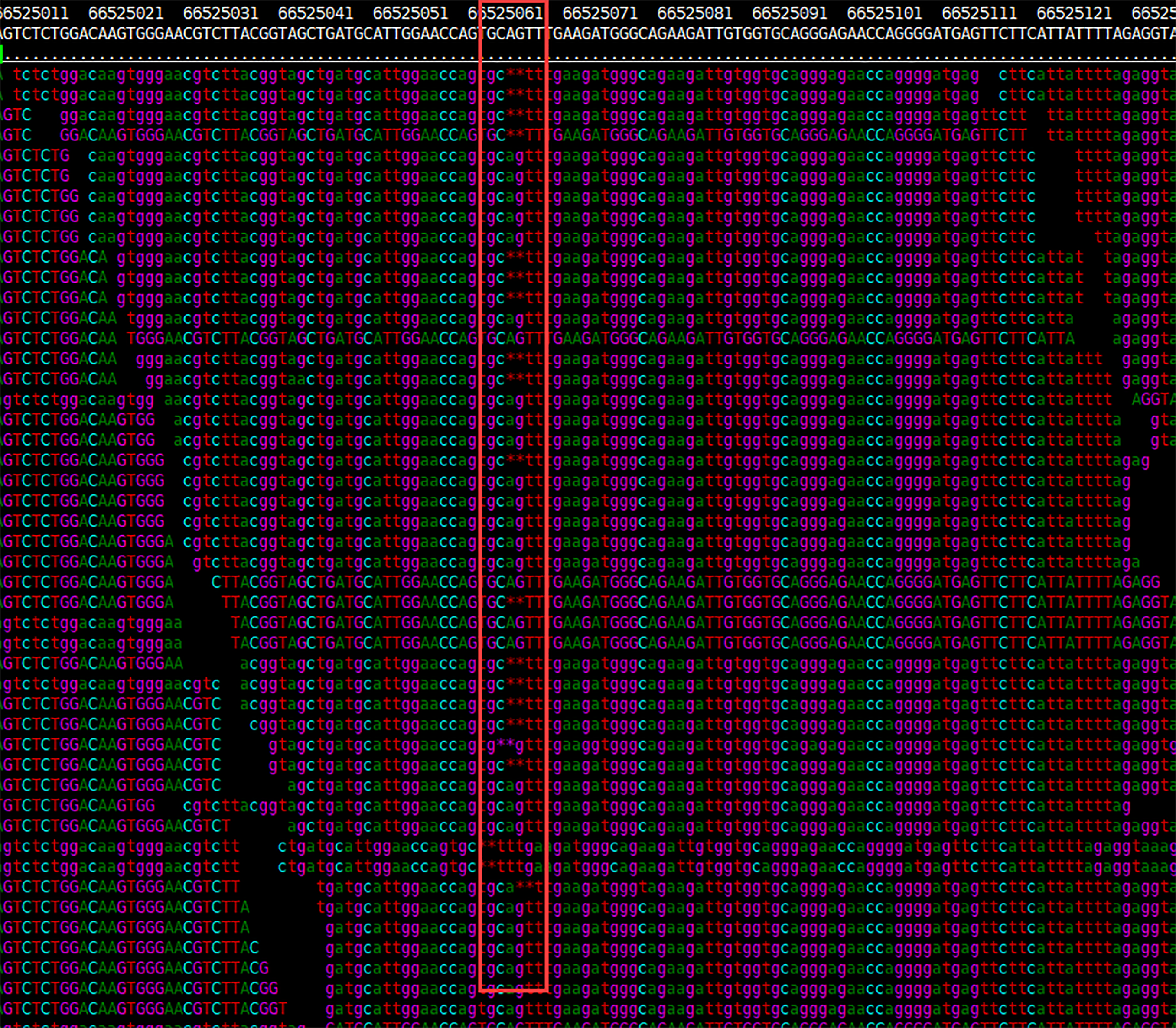

Supplement: Supplementary file 1 [file Image3.TIF]

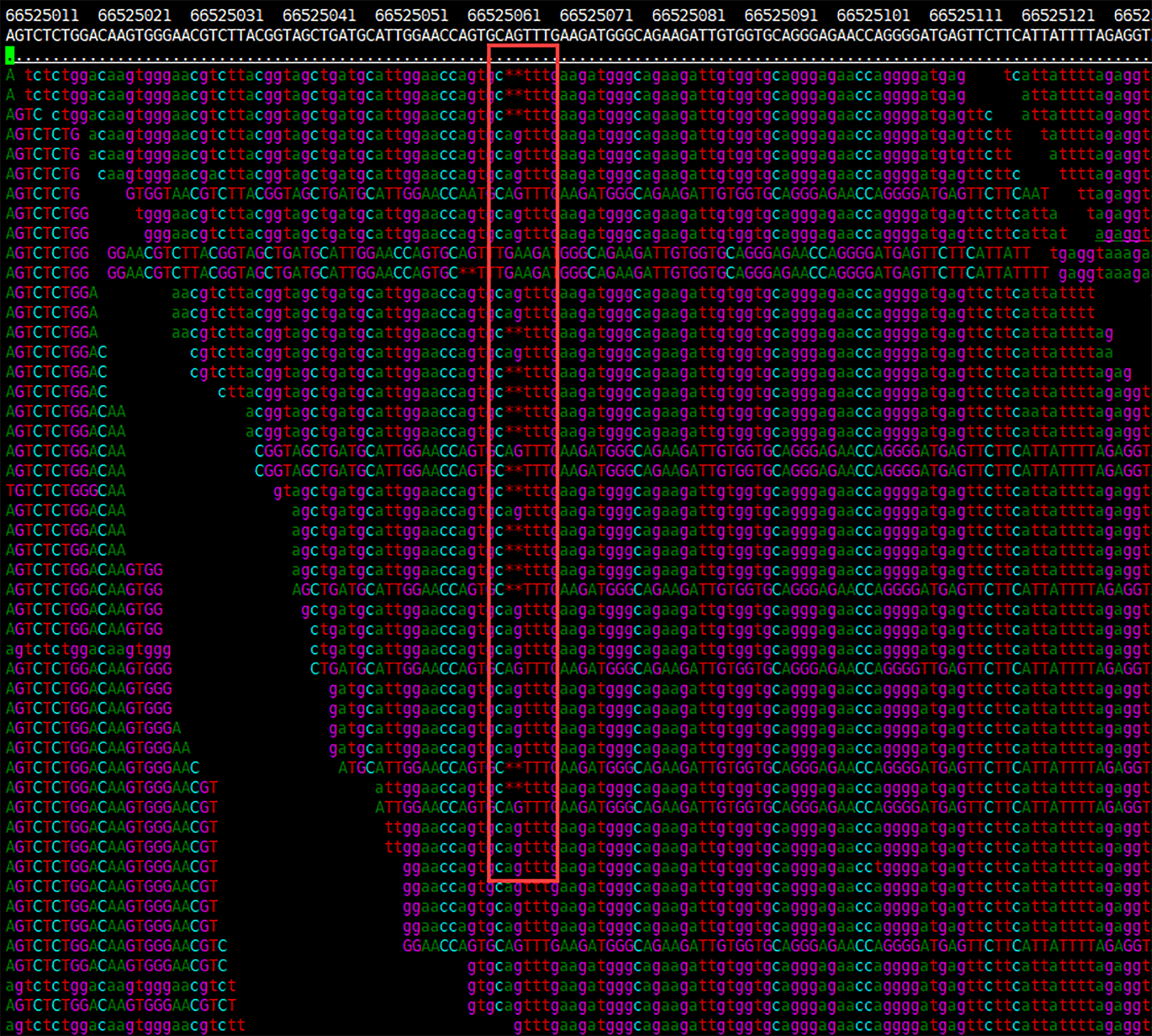

Supplement: Supplementary file 2 [file Image4.TIF]

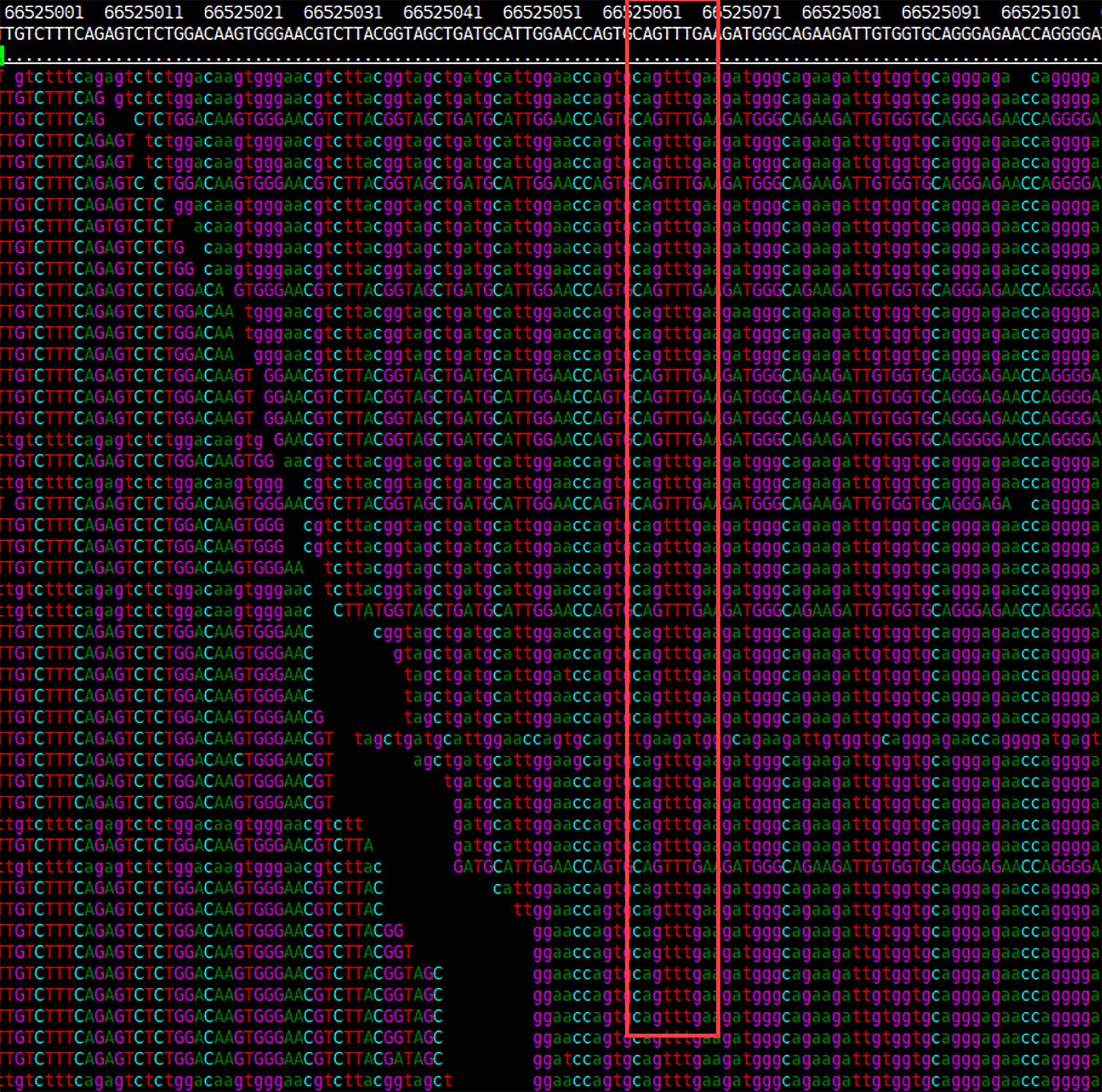

Supplement: Supplementary file 3 [file Image2.TIF]

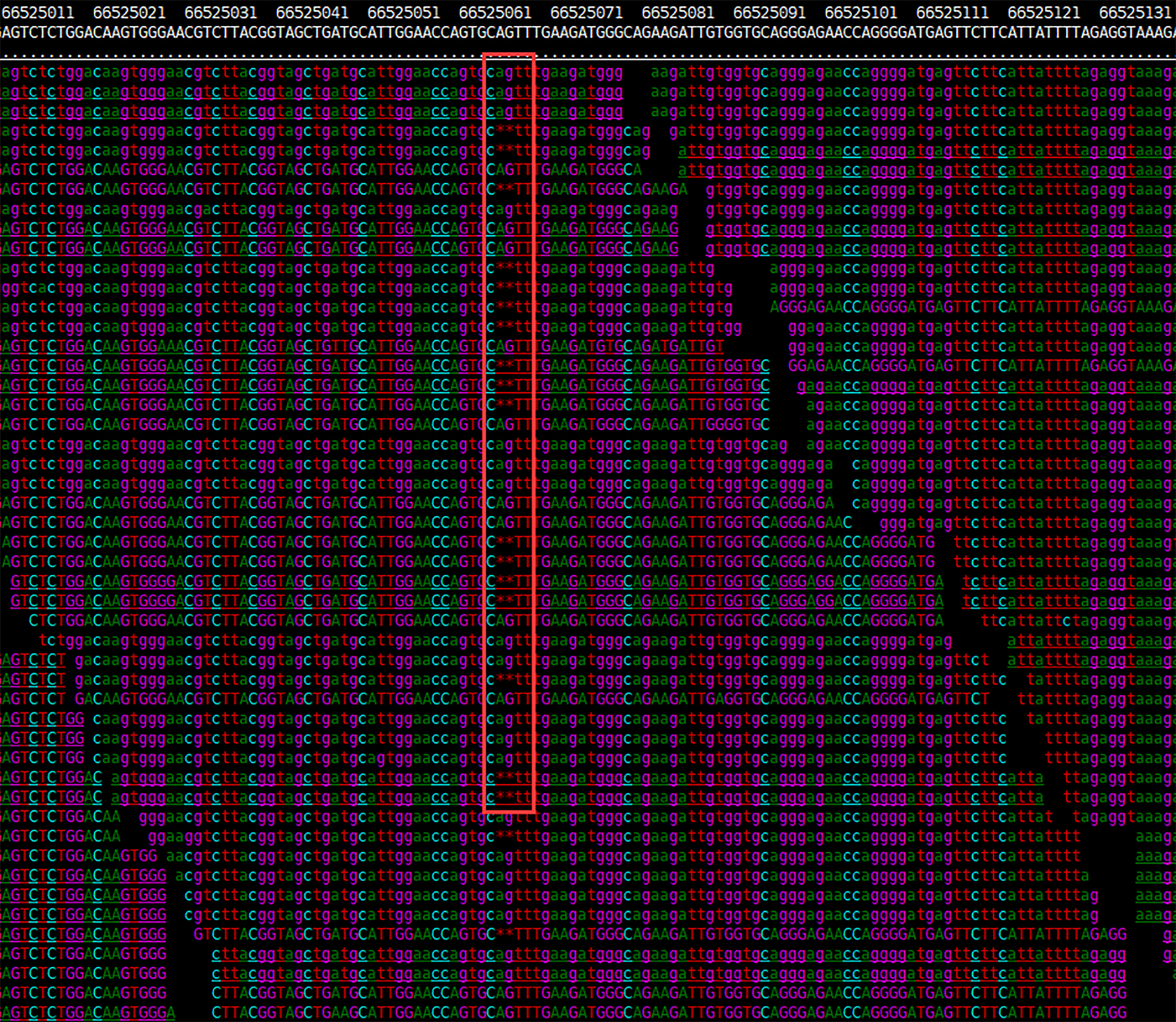

Supplement: Supplementary file 4 [file Image1.TIF]
